# The Utrecht University Honours Program review project: example based scientific publishing training aimed at bachelor medical students

**DOI:** 10.1186/s12909-024-05098-7

**Published:** 2024-02-06

**Authors:** Meye Bloothooft, Helena J. M. Pennings, Marcel A. G. van der Heyden

**Affiliations:** 1 Department of Medical Physiology, Division Heart and Lungs of University Medical Center Utrecht, Yalelaan 50, 3584 CM Utrecht, The Netherlands; 2 Utrecht Center for Research and Development of Health Professions Education, University Medical Center Utrecht, Utrecht, The Netherlands; 3 Department Learning and Workforce Development, Netherlands Organization for Applied Scientific Research (TNO), Soesterberg, The Netherlands

**Keywords:** Scholarly publishing, Bachelor/undergraduate education, Review writing, Skill development

## Abstract

**Introduction:**

Medical undergraduate students receive limited education on scholarly publishing. However, publishing experiences during this phase are known to influence study and career paths. The medical bachelor Honours Program (HP) at Utrecht University initiated a hands-on writing and publishing course, which resulted in nine reviews published in internationally peer reviewed academic journals. We wanted to share the project set-up, explore the academic development of the participating students and determine the impact of the reviews on the scientific community.

**Methods:**

Thirty-one out of 50 alumni completed a digital retrospective questionnaire on for example, development of skills and benefit for their studies and career. Publication metrics of the HP review papers were retrieved from Web of Science.

**Results:**

This hands-on project provides a clear teaching method on academic writing and scholarly publishing in the bachelor medical curriculum. Participants were able to obtain and improve writing and publishing skills.

The output yielded well-recognized scientific papers and valuable learning experiences. 71% of the participating students published at least one additional paper following this project, and 55% of the students indicated the project influenced their academic study and/or career path. Nine manuscripts were published in journals with an average impact factor of 3.56 and cited on average 3.73 times per year.

**Discussion:**

This course might inspire other medical educators to incorporate similar projects successfully into their curriculum. To this end, a number of recommendations with regard to supervision, time investment and group size are given.

**Supplementary Information:**

The online version contains supplementary material available at 10.1186/s12909-024-05098-7.

In the current Dutch academic medical education system bachelor students read, process, and value scientific literature and write reports on it. These learning activities are important in educating future academic clinicians [1–4]. Also, Adebisi commends to publish undergraduate science efforts. Yet, these reports are usually only read by the supervisor/graders [5]. However, several studies have quantified the percentages of medical students that scholarly published during their pre-graduate training, although it is not clear if or how they were involved in the actual submission and publication process. Numbers vary between institutes and countries (Stanford University, 75% [6]; UK, 8.4% [7]; Netherlands, 14.5% [8], for review see Chang and Ramnanan [9]). A recent global analysis on research education among 619 medical students stated that 10.3% of them published a research paper [10]. Again, to what extent these students were involved in the publication process itself has not been reported. Specific numbers on publications by medical students during the bachelor phase of their curriculum could not be retrieved. In contrast to a few examples of graduate teaching [11–14] in the current system we are hardly educating our undergraduate students the skills of how to publish a paper. Which means that valuable lessons on scientific publishing are withheld from these students.

Although only the minority of undergraduate students publish, interestingly, it was found that those who do more often keep publishing in their next career stages compared to non-publishing pre-graduates [15, 16]. Furthermore, several studies found a positive correlation between participating in pre-graduate research and subsequent career positions in academic science [1, 17]. Since these studies show that participating and publishing research in pre-graduate education shows beneficial outcomes for later career stages, it is important that those students who are interested in science get opportunities to commence publishing already in their pre-graduate education.

To provide undergraduate students with the opportunity to not only write, but also learn from publishing their research, over the last decade, the bachelor Honours Program (HP) of Utrecht University faculty of Medicine decided to take a writing assignment (HP review project) one step further. Students were not only taught the scientific writing process, but also received hands-on teaching on publishing. In total 50 students partook in the course, which resulted in nine reviews [18–26] that were published in peer-reviewed scientific journals.

We now wanted to share an overview of the project for others to copy and gain insight in how the students and the scientific community benefitted from the project. To investigate this, we addressed the following research questions:

1. How is the set-up of the project?
2. What were students’ motivations for participating in the HP review project?
3. How and to what extent did students benefit from participating in the HP review project?
4. What is the scientific impact of the published reviews?

## Context and description of the project

The faculty of Medicine of Utrecht University is responsible for the bachelor or undergraduate education (3 years) and master or graduate education (3 years) medical education. After six years of medical school the students are licensed medical doctors [27, 28]. Three out of ten finished medical school students decide to start a PhD trajectory, which is a paid employment in The Netherlands, of generally 3–5 years, after which they obtain their doctoral degree [29].

Each year 304 students enter the medical bachelor. In the second year of the bachelor, students are offered to enrol in a partially extracurricular 2-year Honours Program. Annually, 15–20 students (approximately 90% of the yearly applicants) are selected based on their motivation for research, their study performance during the first year, and their personal capabilities and interests for broadening (*e.g.* medical humanities) and deepening (*e.g.* state of the art research) their studies. HP students may sign up for the HP review project (see Table 1 for learning goals), which is guided by an experienced researcher and teacher, who has ample experience in scholarly publishing. The teacher should have: 1) a teaching degree, 2) at least 10 peer reviewed publications as corresponding author, 3) at least 5 years of scientific writing experience, 4) a PhD degree, 5) experience with teaching/supervising small groups.

**Table 1:** Learning goals and competences of the HP review project

1	Finding and obtaining relevant literature from multiple sources and languages
2	Analysis, interpreting and summarizing scientific content of papers in the context of a research field
3	Using existing primary literature as a basis to produce a review manuscript in the requested format
4	Evaluate scientific journals, their quality indicators and target audience
5	Communicating with Editor-in-Chief of a scientific journal
6	Handling online submission procedures, including open access options
7	Analysis, interpretation and addressing of reviewer comments
8	Adapting a manuscript according to the reviewer comments and writing a response letter
9	Checking of proofs and unambiguous indication of textual changes required

On average, a monthly progress meeting of the participating students and their supervisor was held. During these meetings, the supervisor emphasizes on for example collecting, analysis and summarizing data, structuring text, figure and table construction, journal guidelines, impact factor, the role of peer review, the revision process, copyright vs. open access depending on where the students were in the project. Students could send an e-mail if they had questions or needed help in between meetings. Finally, the supervisor is a cowriter of the manuscript, and provides written and verbal feedback on the text, figures and tables produced by the participating students.

There are no selection criteria for participation in the HP review project, since the students were already selected for the HP. The students can voluntarily participate and in general around 5 students work together on one manuscript and its submission, but the group size can vary, depending on the amount of interest of the HP students per year. In total 50 students participated in the last ten years since the start of the HP review project.

**Fig. 1:**
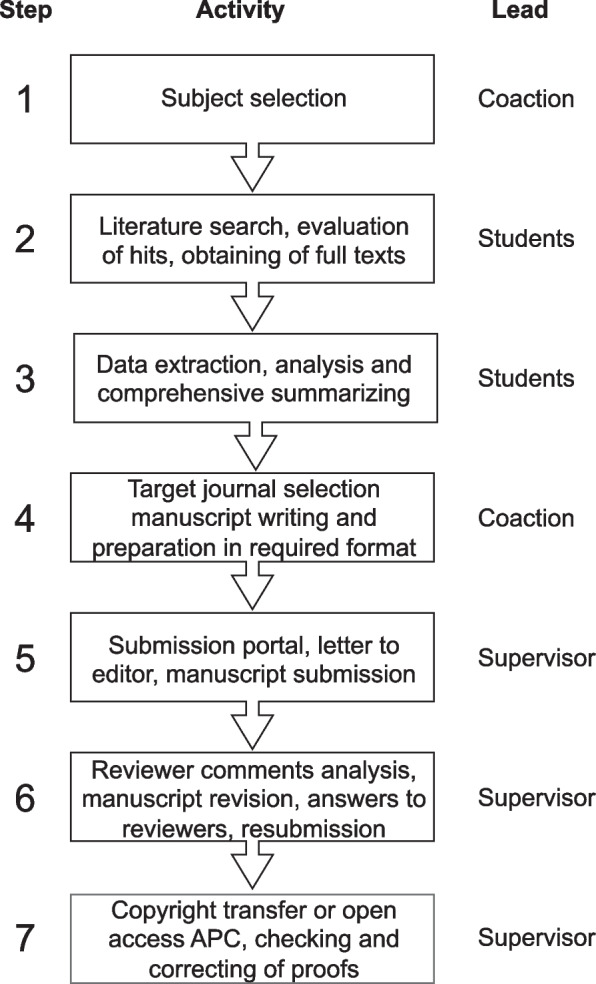
Workflow of the HP review project. The HP review project workflow can be divided in 7 steps, of which the last four are specifically associated with the process of scholarly publishing. All steps have a distinguished separation in task responsibility by either all the students or the supervisor/teacher, although all should be involved

The project involves approximately 18-months of part-time work. The project consists of seven phases, Fig. 1, which have been structured and optimized over the last ten years:

1. The review subject is determined by the participating students and the project supervisor using following criteria: medically oriented, confined, manageable size of primary medical literature (mostly case reports, not more than 50), complexity of the subject should match authors ability to become specialists in 5 months, and appealing.
2. Search strings in different languages are generated and entered in search machines (*e.g.* Pubmed, Google Scholar). Hits are evaluated for suitability and reference lists are screened for additional papers. If necessary, authors or international colleagues are contacted to obtain full text papers.
3. Content of the primary papers is extracted, ordered and summarized (*e.g.* demographics, clinical symptoms, drug dose, treatment) and used as the basis of the manuscript.
4. A list of potential target journals is constructed taking aims and scope, reader audience, research discipline and impact factor into account, and a first option journal is selected. The manuscript is written and checked according to the author guidelines. Also, the order of authors is established: every participating student hands in a ranking list of authors they think will reflect best the investment of the individual authors in the project (time, data analysis, figure producing, writing and reviewing effort, etc.), leaving out their own name. The student with the highest overall ranking gets the number 1 position etc. This typically yields similar lists from all students. This procedure forces students to reflect on their own and others’ contributions. Besides this strict order, in many cases “shared first authorship” has been used.
5. The manuscript is submitted for publication with an adjoining letter to the editor-in-chief.
6. Reviewer comments are read, evaluated, and addressed. Accordingly, the manuscript is revised and resubmitted.
7. Upon acceptance copy-right transfer or open access procedures are followed, and proofs are carefully checked, and corrections are provided.

All students should be involved equally in all steps of the project, also the submission and publication part although the supervisor takes the lead in these steps. The subject selection will take up two sessions—in between the students will do some research on the different proposed topics—and takes one month. The literature search, data extraction, and writing part take up most of the time and in the HP review project take approximately four months each. The submission and publication differ in duration depending on whether the review is accepted directly or is submitted a few times before acceptance and how long it takes before review reports are received. If all goes swiftly this part could be finished in four months. It should be noted that the HP review project was not a full-time project and students only spend a few hours each week on the project.

## Methods

### Design and procedure

This study consists of two parts:

1. Study the students’ benefit and impact of participating in the HP review project. To do so, a retrospective questionnaire study was performed. All 50 students who partook in the HP review project since the start, a decade ago, were invited to participate via e-mail. The questionnaire was digitally distributed via Castor EDC (v2022.5.0.1.). Participants were informed about data security and anonymity via an information letter and had to provide consent for using the data for a publication before they could enter the questionnaire.
2. Obtain insight in the scientific contribution of the published review articles. Metrics (*e.g.* impact factor and number of citations) associated to each article were retrieved via Web of Science.

### Questionnaire

To measure (1) students’ motivation to participate in the project, (2) what students gained from participating in the HP review project and (3) how students were able to use these gained skills in their career, a questionnaire (see Additional file 1: Appendix A) was created. The questionnaire consisted of multiple-choice questions interspersed with open questions in which participants could elaborate on their multiple-choice answer. The questionnaire was made in Dutch, but for this paper the answers have been translated to English.

The questionnaire was distributed to all 50 former students that participated in the HP review project since the start a decade ago via e-mail. Their e-mail addresses were known to the investigator because their e-mailaddress was the same as when they partook in the project, or their e-mailaddress was found publicly online, or via communication with the former participant. Participants received one reminder after a few weeks to fill in the questionnaire.

#### Motivation

Importance of various reasons for participation in the project were measured using six 5-point Likert scale (1 = *very unimportant* to 5 = *very important*) items. For example: “Improving scientific writing”. Followed by an open question for participants to add other reasons for participation.

#### Previous experience with writing and publishing

Previous experience with scientific writing and publishing was measured using 16 items. The first question asked whether the HP review was the first paper they submitted. The other 15 items were rated on a 5-point Likert scale (*1* = *not* to *5* = *a very great deal*). All items started with “How much experience did you have with the following skills before starting the HP review project?” All items are listed in Table 2.

**Table 2:** Different skills used in the project distributed in four different categories for analysis. This item distribution is used for analysis of the students’ experience before (questionnaire question 5), during (questionnaire question 6 and 7) and after (questionnaire question 17, 20 and 28)

Group	Skills
*General skills*	collaborating—adhere to schedule
*Preparation skills*	selecting a subject—making a work schedule—drafting a paper
*Execution skills*	searching literature—interpreting literature—academic writing—producing figures and/or tables—merging written texts—editing a paper—compiling a reference list
*Publishing skills*	selecting a journal for publication—writing a letter to an editor—processing review comments

#### Experience during the project

The experienced difficulty of each task, see Table 2, was measured on a 5-point Likert scale (*1* = *very difficult* to *5* = *very easy*). All items started with “How did you find the application of the following skills during the HP review project?”. The gained experience was measured with a similar question: “To what extent did you gain experience on the following skills during the HP review project?” and a 5-point Likert scale (*1* = *not* to *5* = *a very great deal*). Additional questions were asked to gain insight in the general experience of the project.

#### Collaboration and supervision

To evaluate the experienced collaboration and supervision, participants rated seven items on a 5-point Likert scale (*1* = *very bad* to *5* = *very good*). An example item for collaboration was “decision on the topic”, and for supervision “feedback on written texts”. Both were followed by an open question for participants to elaborate on their experience.

#### Consecutive scientific output

How the project increased their interest in scientific research, how many papers they published after completing the project, and how participants were able to use the skills they gained during the project, was studied in questions like: “To what extent were you able to use gained experience from the HP review project during subsequent publications?”, with a 5-point Likert scale (*1* = *not* to *5* = *a very great deal*) with 15 items, see Table 2.

#### Academic study and working career

To measure whether taught skills were used in their academic study or working career participants rated 15 items, see Table 2 on a 5-point Likert scale (*1* = *not* to *5* = *a very great deal).* The questions on the working career were only answered by the participants that have or have had a working career. We also asked whether participants were enrolled in a PhD program or were planning to and how the HP review project had an impact on their decision to pursue a PhD.

#### Project improvement

Participants were asked about other writing courses they took, and differences compared to the project and if improvements could be made to the HP review project.

### Data analysis

Only complete questionnaires were analyzed. The items in the questionnaire were not designed as scales, but designed to get insight in individual aspects. We performed the analysis on item level to describe the results. To that end, the Means (*M*), Standard Deviations (*SD*), Median and Frequencies corresponding to the items were calculated in IBM SPSS (version 27).

To analyze the data of the 15 skills (Table 2) the items were sorted in 4 different groups, corresponding to different stages of writing and submission.

## Results

### Participants

Out of all 50 former participants that received the questionnaire, 31 (62%) completed the questionnaire. Except for one review (no returned questionnaire) at least 2 and an average of 3.9 contributing authors of the other reviews filled out the questionnaire.

### Motivation for project participation

#### Previous experience

Overall, the participants had limited experience with preparing and executing a review study and writing and submitting a paper (Table 3), only four participants had published a scientific article before.

**Table 3:** Descriptive statistics on the students’ experience on different skills (see Table 2) at different stages of the HP review project. Previous experience (*N* = 31), questionnaire question 5 (1 = not: 5 = a very great deal); Difficulty execution (*N* = 31), questionnaire question 6 (1 = very difficult: 5 = very easy); Gained experience (*N* = 31), questionnaire question 7 (1 = not; 5 = a very great deal); Consecutive papers (*N* = 22) questionnaire question 17 (1 = not: 5 = a very great deal); Academic study (*N* = 31) questionnaire question 20 (1 = not: 5 = a very great deal); Working career (*N* = 14)) questionnaire question 28 (1 = not: 5 = a very great deal)

**Skills**	**Previous experience**			**Difficulty execution**			**Gained experience**			**Consecutive papers**			**Academic study**			**Working career**		
	*mean*	*SD*	*median*	*mean*	*SD*	*median*	*mean*	*SD*	*median*	*mean*	*SD*	*median*	*mean*	*SD*	*median*	*mean*	*SD*	*median*
*general*	3.6	0.6	4	3.7	0.8	4	3.8	0.7	4	3.4	0.9	3	3.4	0.8	4	2.7	1.0	3
*preparation*	2.3	0.8	2	3.1	0.7	3	3.4	0.8	3	3.4	0.9	4	3.0	0.9	3	2.6	1.0	3
*execution*	2.5	0.8	2	3.2	0.8	3	3.7	0.7	4	3.7	0.8	4	3.5	0.9	4	3.0	1.1	3
*publishing*	1.2	0.5	1	2.6	0.8	3	3.0	1.1	3	3.5	1.1	4	2.5	1.2	3	2.3	1.1	3

#### Motivation

The main reasons for students to participate in the project were improvement of their writing performance (*M* = 4.3, *SD* = 0.5, *median* = 4) and wanting to experience the complete process of writing and publishing a paper (*M* = 4.2, *SD* = 1.1, *median* = 5). The topic of the review was of lesser importance for participation (*M* = 2.7, *SD* = 0.8, *median* = 2). Although, by the end of the project most participants gained interest in the topic of their review. Furthermore, in the open questions, two participants stated that “*Good supervision during the writing process*” was also a reason to participate.

### Short- and long-term benefit of participation

#### Previous experience with writing and publishing

**Fig. 2:**
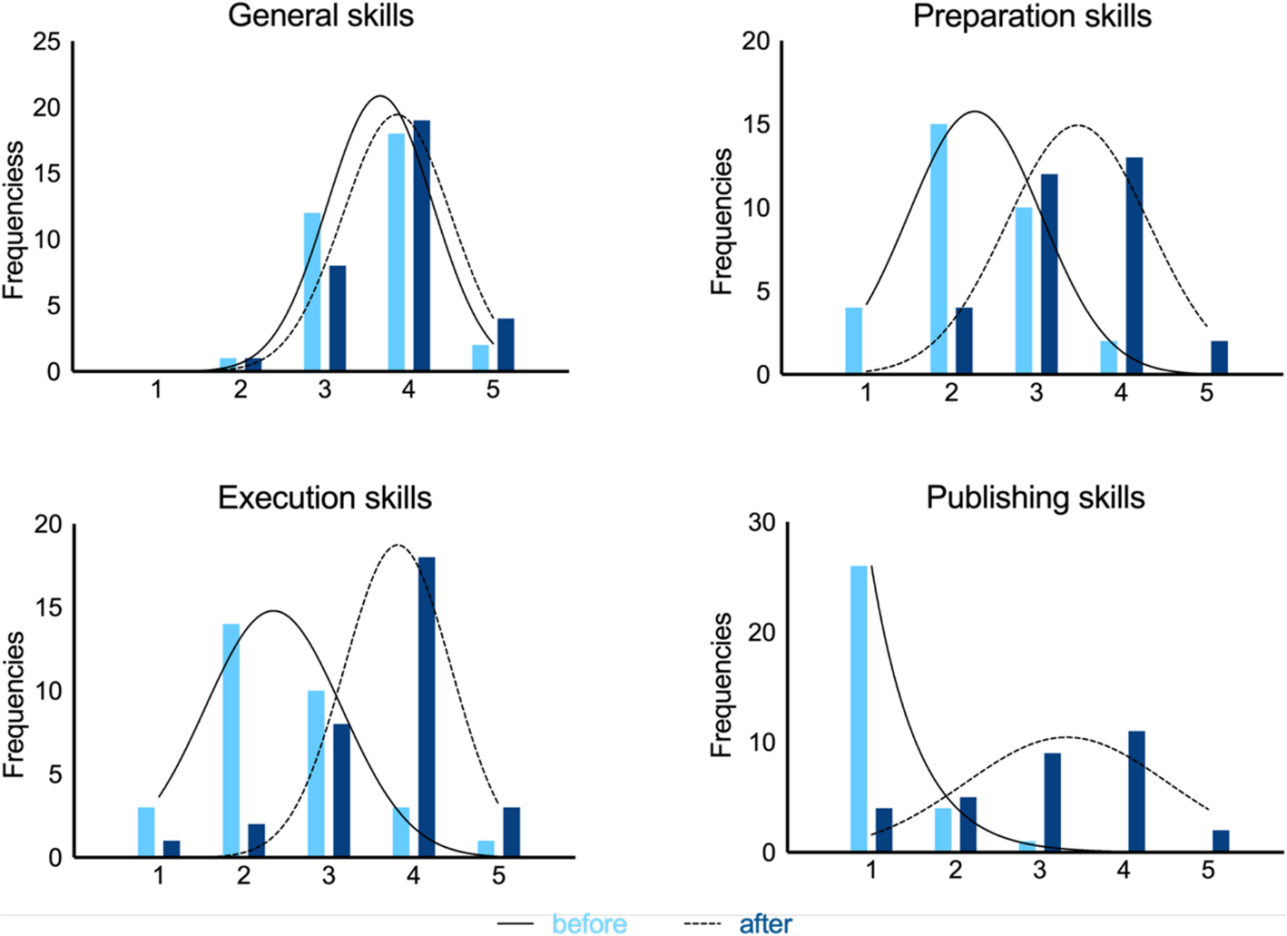
Students’ skills before ”previous experience” and after “gained experience” the project (*N* = 31). 1 = not, 2 = a little, 3 = somewhat, 4 = quite a lot, 5 = a very great deal

During their studies the participants have gained experience on scholarly writing during other assignments (Table 3 “previous experience”, Fig. 2 “before”). Publishing skills were however not trained before, in agreement with the outcomes that show that participants had almost no experience on that aspect. The students were relatively well experienced on their general skills.

#### Experience during the project and skills increase

During the HP review project, participants were able to perform all the different tasks, without experiencing too much difficulty while executing them (all tasks: *M* = 3.1, *SD* = 0.8, *median* = 3, Table 3 “difficulty execution”). Even the execution of publishing skills (*M* = 2.6, *SD* = 0.8, *median* = 3) in which they were not experienced, was not too difficult.

Figure 2 visualizes the differences between the self-reported retrospective previous and gained experience. Compared to their previous experience, participants indicated somewhat higher experience levels in the tasks related to the preparation, execution, and publishing (Fig. 2 and Table 3). The increase in experience was largest in publication skills (∆*M* = 1.8). Some participants indicated not being involved in writing a letter to the editor (*N* = 11) or processing the review comments (*N* = 4), which were important parts of the project. In the end, all participants would participate in the project again.

#### Collaboration and supervision

Participants experienced the collaboration and supervision as good (collaboration: *M* = 3.9, *SD* = 0.7, *median* = 4; supervision: *M* = 4.2, *SD* = 0.8, *median* = 4). In general, the participants state that the division of tasks and communication was well between the students, however some struggled with difference in level of participation between students:

*“Each time it was clear how the tasks were divided and what each person should do before the next meeting.”*, *“We had contact via WhatsApp and saw each other regularly.”* and *“Some students contributed less than others and weren’t as often at the group meetings.”*

One group consisted of 12 students, and that led to some struggles. A subgroup of students took the lead and others did less work:

*“Our group was relatively big, therefore there was a clear core group, and another group that did nearly nothing. That caused some frustration.”* and *“One of the groups took the lead. Which was nice, because that kept the project going, but that made it more difficult to contribute yourself.”*

The supervisor made the setup of the project clear and was willing to help and easy to reach out to. Also, the experience of the supervisor was well appreciated:

“*At the start it was clearly stated what the aim was.”*, *“There was an open atmosphere during the meetings to ask questions, also on the writing process.”* and *“It was nice to have a supervisor with a lot of experience in writing and publishing papers.”*

Also, the students were in charge of the project, which they appreciated to have freedom and their own say in the direction of the project:

*“We were excellently supervised, and the supervisor was always open to asking questions. I liked the balance between letting us figure things out and giving us guidance when needed.”*

#### Consecutive scientific output

**Fig. 3:**
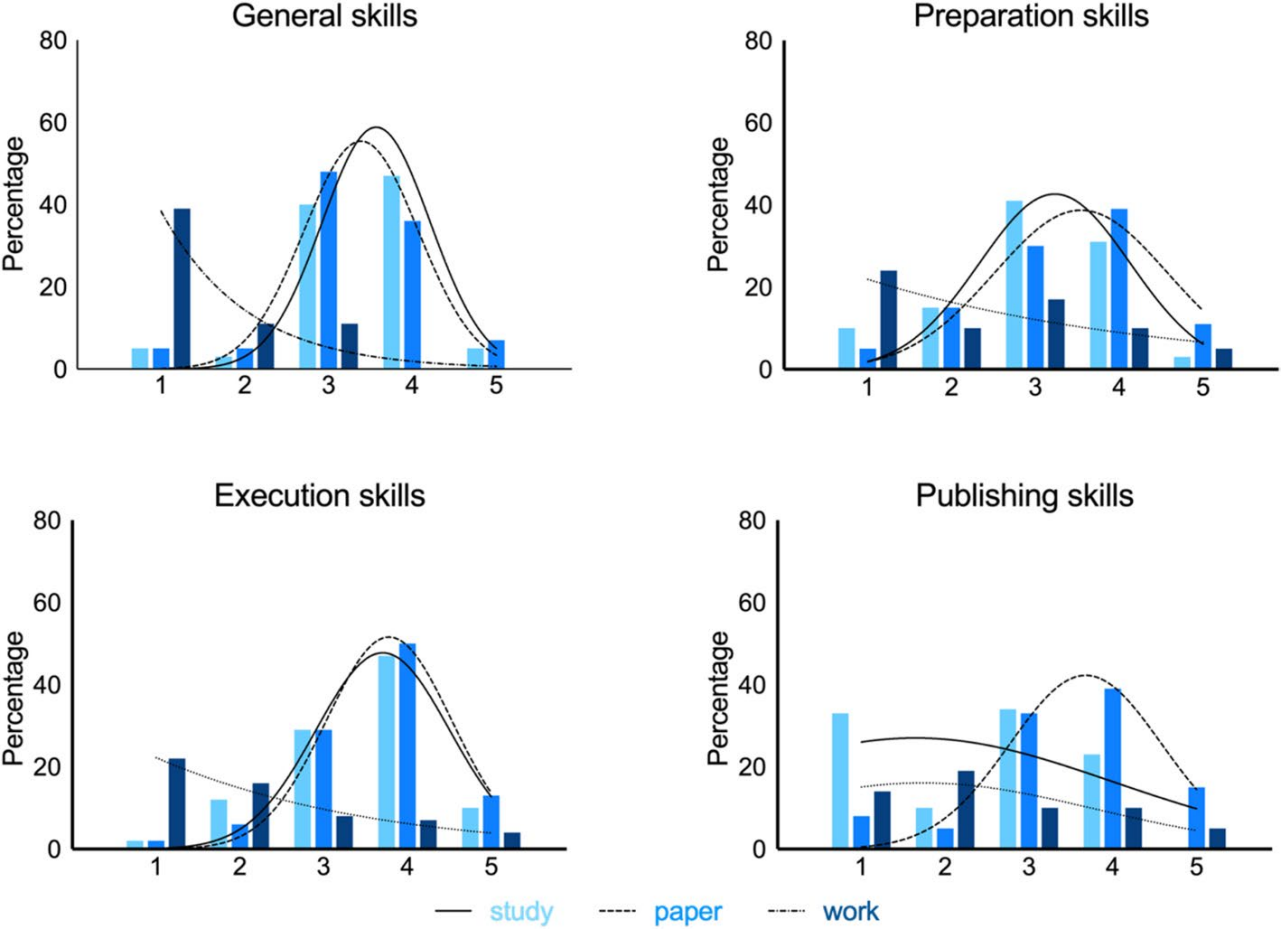
Usefulness of gained skills during next stages in career. Academic study *N* = 31, Consecutive paper(s) *N* = 22, Working career *N* = 14. 1 = not, 2 = a little, 3 = somewhat, 4 = quite a lot, 5 = a very great deal

Seventy one percent (*N* = 22) of the participants indicated having published at least one other paper after the project. During the writing and publishing of consecutive papers, participants indicated being able to use the skills they obtained (Fig. 3 “paper”, Table 3 “consecutive papers”). More specifically, experience in searching literature (*M* = 4.0, *SD* = 0.7, *median* = 4), interpreting literature (*M* = 3.9; *SD* = 0.9; *median* = 4) and scientific writing (*M* = 3.9, *SD* = 0.5, *median* = 4) were deemed most beneficial for writing a subsequent paper.

#### Academic study and working career

For 55% (*N* = 17) of the participants the project influenced their path during their academic studies. In the open questions they indicate their increased interest in science, which resulted in doing (additional) research internships or obtaining grants to do research:

*“It gave me an opportunity to do an internship abroad which led to my current PhD.”* and “*I decided to take a more scientific path for my masters.*”

Participants also stated that they are better in assessing the quality of literature and are better in going through all the steps of publishing:

*“It has helped me finding literature easier and assess it more critically.”* and* ”It gave me an idea of the process of scientific writing and everything that comes with it, collaboration, lay-out, submitting.”*

The participants indicated that they used most obtained skills during the rest of their academic study (Table 3 “academic study”, Fig. 3 “study”). Yet, the publishing skills were not used very much. Especially, searching literature (*M* = 3.9, *SD* = 0.7, *median* = 4), interpreting literature (*M* = 3.7, *SD* = 0.9, *median* = 4), academic writing (*M* = 3.7, *SD* = 0.8, *median* = 4) and collaboration (*M* = 3.7, *SD* = 0.7, *median* = 4) were deemed most relevant during their academic study.

Of the participants, 81% (*N* = 25) indicated that they’re willing to, is doing or finished a PhD. And for most participants the project has had some effect on the decision to pursue a PhD:

*“The publication helped me to obtain a grant for my doctoral studies.”*

Of the participant 45% (*N* = 14) has (had) a working career, of which 35% (*N* = 5) stated the project influenced their career. During their working career (Table 3 “working career, Fig. 3”work”), interpreting literature (*M* = 3.4, *SD* = 1.2, *median* = 4), searching literature (*M* = 3.3, *SD* = 1.2, *median* = 4), academic writing (*M* = 3.0, *SD* = 1.0, *median* = 3) and making a reference list (*M* = 3.0, *SD* = 1.0, *median* = 3) were the most valuable skills. However, not all participants pursued a career in academic science, so some skills, like the publishing skills or selecting a subject for a paper are not used anymore by several participants.

### Project improvement

Forty-five percent (*N* = 14) of the participants have followed an additional writing course. Those courses didn’t deal with the publishing process but focused on writing style/academic writing. Therefore, participants consider the project as an addition to other offered courses. In general students appreciated the project. Some participants suggested improvements, like, more personal feedback moments, or workshops on specific skills, like making figures.

### Scientific impact

To establish the scientific impact of the HP reviews, we collected and analyzed metrics (Table 4). On average, 5 students worked on a manuscript and all nine reviews have been published. The writing and submission process took approximately 16 ± 2 months and the average time between submission and acceptance was 105 ± 82 days. Within the two-year duration of the HP all reviews were submitted and five reviews were accepted. The other reviews were accepted for publication after the HP was finished. Due to waiting time on review reports, editor decisions, and rejections/resubmissions.

**Table 4:** Article metrics associated with the impact of the published reviews

Cohort	Title	Journal (IF^a^)	# HP students	# days submission – acceptance	#Citations (citations/year)^b^
2010–2012	Grayanotoxin poisoning: 'mad honey disease' and beyond [21]	Cardiovasc Toxicol. (3.2)	4	48^c^	82 (7.3)
2012–2014	Barium toxicity and the role of the potassium inward rectifier current [19]	Clin Toxicol (Phila). (3.3)	3	120^c^	55 (5.8)
2013–2015	A Heart too Drunk to Drive; AV Block following Acute Alcohol Intoxication [26]	Chin J Physiol. (1.8)	4	81^c^	10 (1.3)
2014–2016	The toxicology of zinc chloride smoke producing bombs and screens [20]	Clin Toxicol (Phila). (3.3)	4	217	11 (1.6)
2015–2017	Review of case reports on hyperkalemia induced by dietary intake: not restricted to chronic kidney disease patients [24]	Eur J Clin Nutr. (4.7)	4	262	20 (4.1)
2016–2018	Literature Review of Case Reports on Lyme Borreliosis Associated Atrioventricular Conduction Block in Europe between the Years 2000–2017 [23]	Adapt Med. (n.a.)	4	44	not indexed
2017–2019	Nicotine intoxication by e-cigarette liquids: a study of case reports and pathophysiology [22]	Clin Toxicol (Phila). (3.3)	8	58^c^	28 (7.1)
2018–2020	Towards the Development of AgoKirs: New Pharmacological Activators to Study Kir2.x Channel and Target Cardiac Disease [25]	Int J Mol Sci. (5.6)	5	27	4 (1.2)
2019–2021	The clinical course and treatment of black mamba (Dendroaspis polylepis) envenomations: a narrative review [18]	Clin Toxicol (Phila). (3.3)	12	86^c^	3 (1.4)

^a^ 2022 IF

^b^ As of 21st of December 2023

^c^ Review accepted within the 2-year HP

The reviews have been published in peer-reviewed journals with an average impact factor of 3.56, with one journal without an impact factor. After publication the HP review papers were cited 3.73 times per year on average, ranging from highly specialized journals, like the *Journal of Apicultural Research* [30] to general high impact journals, like *the Lancet* [31].

## Discussion

In this study we have explored the contribution that the HP review project had on our bachelor medical students’ careers and in the scientific community.

### Career and scientific impact

In our study, students indicated that many of the skills they were taught during the project were useful in subsequent stages of their career. The participants had a strong interest of pursuing a PhD, 81% is thinking of doing or is doing a PhD, while in The Netherlands around 30% of the medical doctors pursues a PhD [29]. Also, they published many subsequent papers, mainly due to participation in this project. So, students who have a strong interest in science are very suitable to participate in a project like the HP review project as was shown in the previously mentioned correlation between pre-graduate research participation and subsequent positions in academic science [1, 17]. The journal metrics analysis demonstrates that highly motivated bachelor medical students are able to contribute to academic research and produce relevant and well cited publications, having impact on science in general and the medical profession in particular.

### Supervision and peer support

We experienced that successful completion of the project strongly depends on a supervisor trained and well experienced in scholarly publishing to guide the students through the different phases with specific emphasis on the publication skills. Furthermore, the teacher should be motivated and regularly involved in the project, but also provide the students freedom to develop their own interests and skills. On average, during each project a monthly meeting of the participating students and their supervisor for one hour was held. During these meetings, emphasis was given on for example collecting, analysis and summarizing data, structuring text, figure and table construction, journal guidelines, impact factor, the role of peer review, the revision process, copyright vs. open access.

Peer support was seen important during practicing the preparation and execution skills. This is, within each participating student group, specific skills were unevenly distributed among the students. Some were already well accustomed in figure and table preparation, others in statistical analysis and associated software, and for some participants English was a shared native language. During the project, such skills were exchanged between the participants. Also for experience, the group should preferably be between 4 to 6 students, so all students can participate equally and share responsibility for the review.

The subjects of the different HP reviews were sometimes strongly influenced by the supervisor (*e.g.* barium toxicity, development of AgoKirs), while in other cases, the subject was suggested by the student group (*e.g.* alcohol associated heart block, nicotine intoxication, black mamba envenomation). We experienced that the subjects from the students were often strongly influenced by the social context of these young adults (nicotine intoxication, alcohol associated heart block). Also, having a voice in selecting the subject increases intrinsic motivation, although the guidelines for a subject selection as stated before should be considered.

### Dissemination: Setting up a similar project

At Utrecht University, like in many other institutes, honours teaching is regarded as opportunity of developing and experimenting with new education projects [32, 33]. Otto and De Kruif [34] describe several factors that promote diffusion of innovative honours projects into the general curriculum, *i.e.* a classroom environment safe to perform experiments, a teacher community, and a need for institutional support. Thus far, our project experienced a safe environment in which the project could evolve over the years. The supervisor was able to discuss the HP review project with peers, and teachers, in his Utrecht University Honours Teaching program. Finally, the HP review project received financial support.

While writing courses and educational material within academia is established, specific courses and associated course material on the publishing process, like selecting the right journal and keywords, the submission and peer review process, is scarce. However, several journal editors and authors have compiled such specific material to assist prospective authors, which can be used in courses on scholarly publication [35–38] for review see Hardman and Serginson [39]. A similar project as the HP review project was published by Bauler and Jones [12]. In an elective course of one-week second year medical students were taught how to write a case report by experienced academics. After one week the first draft of the case report was finished and upon own motivation the students could publish their case report with the help of the course director. This resulted in 20 published manuscripts in a 4-year time-period. Although a case report is different from a literature review, this other project shows that a writing and publishing course could be implemented in a curriculum in different ways.

If the Utrecht University medical HP program is compared to other HP programs from Dutch medical faculties, they all have a strong focus on research. However, in contrast to our online information, none of the websites of the honours programs from other Dutch Medical Schools, currently specifies the focus on publishing work obtained from courses or internships. Although writing and performing research is embedded in undergraduate (honours) education, the progression to publishing this work lacks. A project such as the HP review project could easily be implemented in extracurricular education, like an honours project. The project can also be implemented in a curriculum next to courses, where students spend a few hours a week on the project for an extended period, such as a couple of months to one or two years. The project could also be offered as a course, wherein students work on (elements of) a review study full time, for several weeks, depending on the assignment. Students can formulate a research question, search strategy and inclusion/exclusion criteria, conduct the literature search, analyze the literature using a codebook, finish the review, find a suitable journal for publishing, write a letter to an editor, and provide each other feedback as reviewers within the time frame of the course. After the course students can decide themselves to publish the review in an actual journal with the aid of the teacher. The teacher can do the submission and have contact with the editor and students can help with processing the review comments, and in case of rejection come up with other journals to publish in. From the decade of experience with the HP review project we formulated some recommendations for a successful dissemination of a similar project.

1. The project should be offered as an elective course, because not all medical students will benefit from hands on knowledge on publication skills in their future career and the project is quite elaborate with a lot of independence.
2. The duration of the project should fit the regular writing-publication time frame, therefore a course duration spread out over 18 months (26 full time days, approximately 7.5 ECTS) would be optimal. But a full-time course could also be implemented, however, the publication process might take a long time due to waiting time on review reports for example. So, the publication should be finished after the course.
3. The project requires supervision by teachers well experienced in scholarly publishing including its financial support.
4. A group size of 4–6 students is perceived as optimal.

Obviously, conditions 2 and 3 are most challenging to implement into an existing curriculum but would fit an (honours) program with a duration of 2–3 years.

## Limitations

All former participants that have written a review were asked to fill in the questionnaire. Although the response rate of completed questionnaires was good, the number of respondents (31) remains relatively low. Furthermore, some of the participants participated a decade ago to the HP review project. They also mentioned that for some questions it was hard to remember what the project was like back then and they are also more progressed in their career and may respond differently to some of the questions compared to if they would have filled in the questionnaire when they were student. This could have resulted in some bias.

## Conclusion

All reviews that were written in the last decade were published and well cited. Therefore, this project is an addition to the scientific community. During the HP review project, participants learned new skills, which were useful throughout their following career. The project really had an impact on quite a few participants, in obtaining grants or their increased interest in science. Even if the impact wasn’t that big, all participants appreciated the project and would happily participate again. So, both the scientific community and students benefit from such a project. It would be recommended that more universities would add projects like this to their curriculum, to disseminate bachelor students’ work to a larger scientific community.

### Supplementary Information

**Additional file 1: Appendix A.** HP review questionnaire

## Data Availability

The datasets used and/or analysed during the current study are available from the corresponding author on reasonable request.
