# Supplementary material for: The Utrecht University Honours Program review project: example based scientific publishing training aimed at bachelor medical students
Source: BMC Med Educ. 2024 Feb 6;24:115. doi: 10.1186/s12909-024-05098-7 (PMC10848494; doi:10.1186/s12909-024-05098-7)
Supplement: Supplementary file 1 — Additional file 1: Appendix A. HP review questionnaire [file 12909_2024_5098_MOESM1_ESM.docx]

**The Honours Program review project: example based scientific publishing training aimed at bachelor medical students.**

BMC Medical Education

Meye Bloothooft^1^, MSc, Helena J.M. Pennings^2,3^, PhD, Marcel A.G. van der Heyden, PhD^1^

1. Department of Medical Physiology, Division Heart and Lungs of University Medical Center Utrecht, Utrecht, The Netherlands

2. Utrecht Center for Research and Development of Health Professions Education, University Medical Center Utrecht, Utrecht, The Netherlands

3. Netherlands Organization for Applied Scientific Research (TNO), Department Human Behavior and Training, Soesterberg, The Netherlands

E-mail: m.a.g.vanderheyden@umcutrecht.nl

**Appendix A**

HP review questionnaire

With the first questions we will go into your motivation and experience before you started the HP review project.

1. Which review did you participated in?

Options: Grayanotoxin poisoning, Barium toxicity, AV block and alcohol intoxication, Toxicology zinc chloride smoke bombs, Dietary induced hyperkalemia, Lyme and AV block, Nicotine intoxication, AgoKirs, Black mamba

1. Was the HP review your first paper you have written and submitted for publication?

Options: yes, no

1. To what extent were following reasons important for you to join the HP review project?

Subjects: improving academic writing, going through the entire publication process, a publication for your CV, working together with fellow students, building a professional network, the topic of the review

Options: Very unimportant, Unimportant, Neither unimportant nor important, Important, Very important

1. Did you have any other reasons to participate in the HP review project?
2. How much experience did you have with the following skills before you participated in the HP review project?

Subjects: selecting a subject, drafting a paper, making a work schedule, collaborating, searching literature, interpreting literature, academic writing, making figures and/or tables, merging written texts, editing a paper, making a reference list, selecting a journal for publication, writing a letter to an editor, processing review comments, adhere to schedule

Options: Not, A little, Somewhat, Quite a lot, A very great deal

The following questions will address your experiences during the HP review project.

1. How did you find the application of the following skills during the HP review project?

Subjects: selecting a subject, drafting a paper, making a work schedule, collaborating, searching literature, interpreting literature, academic writing, making figures and/or tables, merging written texts, editing a paper, making a reference list, selecting a journal for publication, writing a letter to an editor, processing review comments, adhere to schedule

Options: Very difficult, Difficult, Neither difficult nor easy, Easy, Very easy

1. To what extent did you gain experience on the following skills during the HP review project?

Subjects: selecting a subject, drafting a paper, making a work schedule, collaborating, searching literature, interpreting literature, academic writing, making figures and/or tables, merging written texts, editing a paper, making a reference list, selecting a journal for publication, writing a letter to an editor, processing review comments, adhere to schedule

Options: Not, A little, Somewhat, Quite a lot, A very great deal

The following questions will address your general experience of the HP review project.

1. To what extent did the interest in the subject of your review increase?

Options: Not, A little, Somewhat, Quite a lot, A very great deal

1. To what extent did the duration of the project correspond to the expected duration before the start of the project?

Options: Not, somewhat, completely

1. Would you do the project again?

Options: Yes, No

The following questions will address the collaboration with fellow students during the HP review project.

1. How did you experience the collaboration with other students on the following topics during the HP review project?

Subjects: general contact, number of meetings, agreements on the division of tasks and schedule, decision on the topic, merging texts to a paper, asking help with problems or questions, providing feedback

Options: Very bad, Bad, Neither bad nor good, Good, Very good

1. Could you tell more on how you experienced the collaboration with other students?

With the following questions we address the supervision during the HP review project.

1. How did you experience the supervision on the following topics during the HP review project?

Subjects: instruction on the project, accessibility of the supervisor, number of meetings, guidance of the project, subject specific input, feedback on written texts, communication on the status of the submitted review

Options: Very bad, Bad, Neither bad nor good, Good, Very good

1. Could you tell more on how you experienced the supervision?

The following questions will address the gained experience during the HP review project and how you used that experience in subsequent publications.

1. To what extent did writing and publishing the review increased your interest in performing scientific research?

Options: Not, A little, Somewhat, Quite a lot, A very great deal

1. How many papers did you publish after the HP review project?
2. To what extent were you able to use gained experience during the HP review project during subsequent publications?

Subjects: selecting a subject, drafting a paper, making a work schedule, collaborating, searching literature, interpreting literature, academic writing, making figures and/or tables, merging written texts, editing a paper, making a reference list, selecting a journal for publication, writing a letter to an editor, processing review comments, adhere to schedule

Options: Not, A little, Somewhat, Quite a lot, A very great deal

The following questions will address your gained experience during the HP review project and how you used that experience during your academic studies.

1. Did writing of the review helped you during your academic studies?

Options: Yes, No

1. How did the HP review project help you during your academic studies?
2. To what extent were you able to use following gained experiences during the HP review project during your academic studies?

Subjects: selecting a subject, drafting a paper, making a work schedule, collaborating, searching literature, interpreting literature, academic writing, making figures and/or tables, merging written texts, editing a paper, making a reference list, selecting a journal for publication, writing a letter to an editor, processing review comments, adhere to schedule

Options: Not, A little, Somewhat, Quite a lot, A very great deal

1. Did the HP review project have impact on your subsequent study path?

Options: Yes, No

1. Where did the HP review project have impact on?

Following question will go into you experience after the HP review project and how you used that experience during your working career.

1. Did you start a PhD or are you planning to?

Options: Yes, No

1. To what extent did the HP review project have effect on the decision to start a PhD?

Options: None, A little, Somewhat, Quite a lot, A very great deal

1. Do you have a working career at the moment, or have you had one?

Options: Yes, No

1. Did the HP review have impact on your working career?

Options: Yes, No

1. What aspects did the HP review project have impact on?
2. To what extent were you able to use following gained experiences during the HP review project during your working career?

Subjects: selecting a subject, drafting a paper, making a work schedule, collaborating, searching literature, interpreting literature, academic writing, making figures and/or tables, merging written texts, editing a paper, making a reference list, selecting a journal for publication, writing a letter to an editor, processing review comments, adhere to schedule

Options: Not, A little, Somewhat, Quite a lot, A very great deal

1. Did you follow any other writing courses?

Options: Yes, No

1. Did that writing course also dealt with the whole writing till publishing trajectory?

Options: Yes, No

1. Did you miss any parts in the HP review project that were dealt with in the other writing course?

Options: Yes, No

1. What were the parts that were dealt with in the other course, but not during the HP review project?
2. Are there any parts on which the HP review project could be improved?
3. Do you have any other comments?
